# Targeting of CAT and VCAM1 as Novel Therapeutic Targets for DMD Cardiomyopathy

**DOI:** 10.3389/fcell.2021.659177

**Published:** 2021-04-01

**Authors:** Bin Li, Weiyao Xiong, Wen-Miin Liang, Jian-Shiun Chiou, Ying-Ju Lin, Alex C. Y. Chang

**Affiliations:** ^1^Department of Cardiology and Shanghai Institute of Precision Medicine, Ninth People’s Hospital, Shanghai Jiao Tong University School of Medicine, Shanghai, China; ^2^Department of Health Services Administration, China Medical University, Taichung, Taiwan; ^3^School of Chinese Medicine, China Medical University, Taichung, Taiwan; ^4^Genetic Center, Proteomics Core Laboratory, Department of Medical Research, China Medical University Hospital, Taichung, Taiwan

**Keywords:** DMD cardiomyopathy, CAT, VCAM1, CHM herbs, natural compounds, hiPSC-CMs

## Abstract

Duchenne muscular dystrophy (DMD) related cardiomyopathy is the leading cause of early mortality in DMD patients. There is an urgent need to gain a better understanding of the disease molecular pathogenesis and develop effective therapies to prevent the onset of heart failure. In the present study, we used DMD human induced pluripotent stem cells (DMD-hiPSCs) derived cardiomyocytes (CMs) as a platform to explore the active compounds in commonly used Chinese herbal medicine (CHM) herbs. Single CHM herb (DaH, ZK, and CQZ) reduced cell beating rate, decreased cellular ROS accumulation, and improved structure of DMD hiPSC-CMs. Cross-comparison of transcriptomic profiling data and active compound library identified nine active chemicals targeting ROS neutralizing Catalase (CAT) and structural protein vascular cell adhesion molecule 1 (VCAM1). Treatment with Quecetin, Kaempferol, and Vitamin C, targeting CAT, conferred ROS protection and improved contraction; treatment with Hesperidin and Allicin, targeting VCAM1, induced structure enhancement via induction of focal adhesion. Lastly, overexpression of CAT or VCAM1 in DMD hiPSC-CMs reconstituted efficacious effects and conferred increase in cardiomyocyte function. Together, our results provide a new insight in treating DMD cardiomyopathy via targeting of CAT and VCAM1, and serves as an example of translating Bed to Bench back to Bed using a muti-omics approach.

## Introduction

Duchenne muscular dystrophy (DMD) is an X-linked genetic disease affecting ∼1:3500 to 1:5000 males worldwide, caused by loss of dystrophin expression (Lapidos et al., 2004; Verhaart and Aartsma-Rus, 2019). Dystrophin, a protein that provides anchorage of intracellular cytoskeleton to extracellular matrix via formation of the dystrophin glycoprotein complex (DGC), confers mechanical stability, cellular signaling and cellular integrity in skeletal and cardiac muscles (Koenig et al., 1988; Petrof et al., 1993; Talsness et al., 2015). Patients born with this disease experience progressive muscle loss leading to muscle weakness, and by their late 20 s, succumb to dilated cardiomyopathy and respiratory failure. DMD cardiomyopathy is progressive and the present cardiac phenotypes include: arrhythmia, ECG abnormalities, diastolic dysfunction, fibrosis, gradual ventricular dilation, systolic dysfunction, and end-stage heart failure (Finsterer and Cripe, 2014; Yucel et al., 2018). As a leading cause of death in DMD patients, there is an urgent need to develop effective therapies for treating DMD cardiomyopathy and prevent the onset of heart failure.

Currently, Duchenne patients are prescribed with steroids (to slow down muscle deterioration) and general β-blockers (to prolong cardiac function) (Birnkrant et al., 2018). Chronic steroid usage is associated with significant side effects including increase in weight, growth arrest, and the increase risk of bone fracture (Angelini, 2007; Angelini and Peterle, 2012). Besides standard treatment, Chinese herbal medicine (CHM) has always been used as an adjunct therapy for diseases in Taiwan’s healthcare system since 1995. CHM has also been used for physical frailty and showed anti-muscular atrophy effects in mouse models (Moorwood et al., 2011; Zhang et al., 2014; Zeng et al., 2019).

Previous studies have showed human induced pluripotent stem cell (hiPSC)-derived cardiomyocytes (CMs) from DMD patients (DMD hiPSC-CMs) as a valid model to study mechanisms and treatment approaches for DMD cardiomyopathy (Lin et al., 2015; Chang et al., 2018). In this study, using DMD hiPSC-CMs as a validation platform to find the effective natural compounds in CHMs, we validate and identify active components of these CHM herbs. Cross-comparison of transcriptomic profiling data and active compound library identifies nine active chemicals targeting Catalase (CAT) and vascular cell adhesion molecule 1 (VCAM1). Through treating DMD hiPSC-CMs with these active chemicals, we show that Quecetin, Kaempferol, and Vitamin C confer increased CAT activity and were able to decrease H_2_O_2_ and increase DMD hiPSC-CMs contraction function; Hesperidin and Allicin induced VCAM1 expression in cytosol and enhanced sarcomere structure. Overexpression of either CAT or VCAM1 resulted in restoration of DMD hiPSC-CMs function. Together, our data provide an efficient Bed to Bench back to Bed translational avenue for DMD drug development.

## Materials and Methods

### Ethics

This study was approved by the Human Studies Committee of China Medical University Hospital, Taichung, Taiwan [approval number: CMUH107-REC3-074(CR1)]. All protocols using human iPSC were reviewed and approved by the Ethics Review committee at Ninth People’s Hospital, Shanghai Jiao Tong University School of Medicine (2018-207-K32).

### Cell Culture and Cardiac Differentiation

Human iPSCs were cultured on Matrigel (Corning) coated plates with Nutristem hPSC XF medium (Biological Industries) and passaged every 2–3 days by performing a 1:6 dilution. At 70–90% confluency, hiPSCs differentiation was induced to generate beating CMs as described previously (Chang et al., 2018). Briefly, hiPSC were treated with 4–6 μM CHIR-99021 (SelleckChemicals) for 2 days, followed by a Wnt inhibitor IWR-1 treatment (5 μM; Sigma) for another 2 days, in RPMI 1640 medium supplemented with B27 minus insulin (Thermo Fisher Scientific). On day 5, medium were changed to fresh RPMI 1640 medium supplemented with B27 minus insulin for 2 days and switched to RPMI 1640 medium supplemented with B27 until day 10. HiPSC-CMs were then purified using a metabolic-selection medium which consisted of RPMI 1640 without glucose, B27 supplement (Life Technology) and 4 mM of sodium DL-lactate (Sigma). Medium was changed every 2 days for the maintenance of cardiomyocytes. DMD and Healthy hiPSC lines were characterized in Supplementary Table 1.

### Chinese Herbs Powder Extraction Preparation

Chinese herbal medicine stock solution was generated by dissolving 1 g Chinese herb extract (Jiangyin Tianjiang Pharmaceutical Co. Ltd) in 40 ml sterilized water and shook overnight at room temperature. The solution was centrifuged at 3000 rpm for 30 min. The supernatant was then filtered using 0.22 μm filter, aliquoted, and stored at –20°C. Cell were treated using 0%/2%/5%/10% v/v concentration prior to downstream assays.

### Real Time-Quantitative PCR (RT-qPCR)

Total RNA was extracted using TransZol Up Plus RNA Kit (TransGen Biotech) according to the manufacturer’s instructions. Hundred nanogram RNA was used to generate cDNA by using the AMV Reverse Transcription System (TOYOBO). Real-time quantitative PCR was carried out with LightCycler ^@^ 480 II using a ChamQ Universal SYBR qPCR Master Mix (Vazyme) with GAPDH as a reference. Expression was quantified using ΔΔ^Ct^ method and expressed as fold enrichment. Primer sequences are listed in Supplementary Table 2.

### Bioinformatic Analysis

For the cross comparison, we first generated a list of differentially regulated genes in DMD hiPSC-CMs compared to WT hiPSC-CMs. Next, we accessed three publicly available CHM active compound databases for this study (SuperTCM^1^; SymMap^2^; TCMSP^3^). Active compounds and its gene targets for DaH, ZK, and CQZ were manually searched, and a list of active compounds to target genes was compiled. A Venn comparison was performed, and the results are shown below and now supplemented as a Supplementary Table 3.

### Active Compounds Treatment

All concentrations of the active compounds were derived from previous published studies and DMD hiPSC-CMs were treated for 5 days prior to functional assay. Specifically: Quercetin, Naringenin, Kaempferol, and Hesperidin were used at 10 μM (Gutiérrez-Venegas et al., 2014; Denaro et al., 2021); Diosgenin at 200 ng/mL (Zhao et al., 2018); D-lactic acid at 10 μM (Pohanka, 2020); Vitamin C at 10 μM (Parra-Flores et al., 2019); Oxalic acid (10 μM) (Liu et al., 2018); and Allicin at 10 μM (Xiang et al., 2020).

### Cardiomyocyte Contractility Assay

Single hiPSC-CMs were seeded on micropatterns 3 days prior to assay as previously described (Chang et al., 2020). Analysis of spontaneous beating of hiPSC-CMs was performed using video microscopy. Cardiomyocytes were maintained at 37°C and 5% CO_2_ to keep physiologic conditions and bright-field videos (captured at 60 fps) were acquired using an Olympus IX83 microscopy. Contraction speed and beating frequency were extrapolated using an established Conklin method algorithm. Quantification of hiPSC-CMs contraction and beating rate was calculated using Matlab-based motion-tracking software as previously described (Huebsch et al., 2015).

### Measurement of Intracellular ROS and Mitochondrial ROS

Total ROS levels in cardiomyocytes were measured using the Cellrox Oxidative Stress dye per manufacture’ instructions (Invitrogen). Mitochondrial ROS levels in cardiomyocytes were measured using a Mitosox Red Mitochondrial Superoxide Indicator (Invitrogen). Briefly, hiPSC-CMs were incubated with Cellrox/Mitosox and Hoechst 33342 for 30 min at 37°C, washed once with PBS, and the fluorescent signal intensity was measured with Operetta CLS High Content Imaging System (PE) using a 20 × numerical aperture.

### Immunofluorescence Staining

Day 30 cardiomyocytes grown on Matrigel covered coverslips were washed with PBS and fixed in 4% formaldehyde in PBS for 10 min at room temperature. After three PBS washes, cells were permeabilized and blocked with staining solution (0.1% Triton X-100 and 20% FBS in PBS) for 1 h at room temperature. Rabbit monoclonal anti- phospho-histone H2AX (Ser 139) (1:1000 dilution, Santa Cruz), mouse monoclonal anti-cTnT (1:400 dilution, Abcam), rabbit polyclonal anti-ACTN2 (1:200 dilution, proteintech), mouse monoclonal anti-Integrin beta 1 (1:300 dilution, Abcam), or rabbit polyclonal anti-VCAM1 (1:200 dilution, Proteintech) antibodies were diluted in staining solution and samples were incubated overnight at 4°C. Cells were washed in staining solution three times for 10 min each at room temperature and incubated with Alexa Fluor 594 or 488 conjugated secondary antibodies (1:1000 dilution, Invitrogen) at room temperature for 1–2 h. Samples were then washed three times with staining solution and then incubated with PBS containing 4′,6-diamidino-2-phenylindole (DAPI) for 10 min and mounted with VECTASHIELD (VECTOR). Image acquisition was performed using a Zeiss LSM880 microscope and analyzed using ZEN software (Zeiss).

### RNA-Sequencing

RNA-sequencing was performed by GENEWIZ company using the Illumina HiSeq instrument. Total RNA from day 30 WT and DMD hiPSC-CMs were isolated using the TRIzol Reagent (Life Technologies). One microgram total RNA (RIN value above 7) was used for library construction using the NEBNext^®^ Ultra^TM^ RNA Library Prep Kit for Illumina^®^.

Libraries were multiplexed and loaded onto Illumina HiSeq instrument (Illumina). Sequencing was carried out using a 2 × 150 bp paired-end (PE) configuration; image analysis and base calling were conducted by the HiSeq Control Software (HCS) + OLB + GAPipeline-1.6 (Illumina) on the HiSeq instrument. Differential expression analysis was carried out using the DESeq Bioconductor package (Anders and Huber, 2010). After adjusting with Benjamini and Hochberg’s approach for accounting the false discovery rate, *P*-value of *p* < 0.05 was deemed statistically significant and was used to identify differential expressed genes.

### Transmission Electron Microscopy

HiPSC-derived cardiomyocytes were fixed using 2.5% glutaraldehyde solution for 2 h at room temperature inside a fume hood, washed three times with PBS, and post-fixed with 1% osmium tetroxide for 4 h. Next, samples were washed with PBS for three times, then dehydrated in pure ethanol two times (each for 30 min) and infiltrated with ethanol: epoxy 812(2:1,1:1,1:2, each for 30 min). Next, samples were embedded with Epon resin, sectioned at 70 nm thickness. The thin sections were mounted onto formvar-coated copper grids, counterstained with 3% uranyl acetate in 70% methanol and 30% water for 7 min, followed by lead citrate for 3 min. Micrographs were captured using a FEI (Tecnai G2 Spirit 120 kV) electron microscope.

### IMP and EFP Measurements

Impedance (IMP) and extracellular field potential (EFP) measurements were conducted according to Nanion’s standard procedures for the CardioExcyte 96. Briefly, CardioExcyte 96 Sensor Plates (Nanion Technologies) were pre-coated overnight with 1% Fibronectin phosphate-buffered saline (PBS) solution. Cardiomyocytes were seeded at 50,000 viable cells per well. Extracts/small compounds and hiPSC-CMs were incubated for 5 days prior to data acquisition. Medium was exchanged every other day. On the day of measurement, fresh medium was changed 2 h prior to assay. DMSO (0.01%) on the same plate was used as vehicle control. Data was analyzed using DataControl 96 software (Nanion).

### Lentivirus Production and Gene Overexpression in DMD hiPSC-CMs

CAT or VCAM1 cDNA was PCR amplified and cloned into the target gene lentiviral plasmid (pLVX-ZsGreen1 backbone). HEK293T cells were plated in a 10 cm dish and transfected with packaging plasmids using Lipofectamine 2000 according to the manufacturer’s protocol (Life Technologies). The supernatant of transfected cells was collected after 24 h. After centrifuged at 6000 *g* for 15 min, the supernatant containing viruses was concentrated using Lenti-X Concentrator (Clontech). DMD hiPSC-CMs were seeded into 6-well plates and infected with lentiviruses for 24 h and then cultured 5 days prior to functional assay.

### Statistical Analysis

All experimental data are presented as mean ± SD. Statistical significance between two groups was determined using two-tailed Student’s *t*-test. For multiple group comparison, One-way ANOVA with Tukey’s multiple comparison was used. *p*-value less than 0.05 was considered statistically significant. Data were analyzed and represented with GraphPad Prism. *^∗^P* < 0.05, *^∗∗^P* < 0.01, and *^∗∗∗^P* < 0.001.

## Results

### Identification of 4 CHM Herbs in the Treatment of Muscular Dystrophic Patients in Taiwan

**FIGURE 1:**
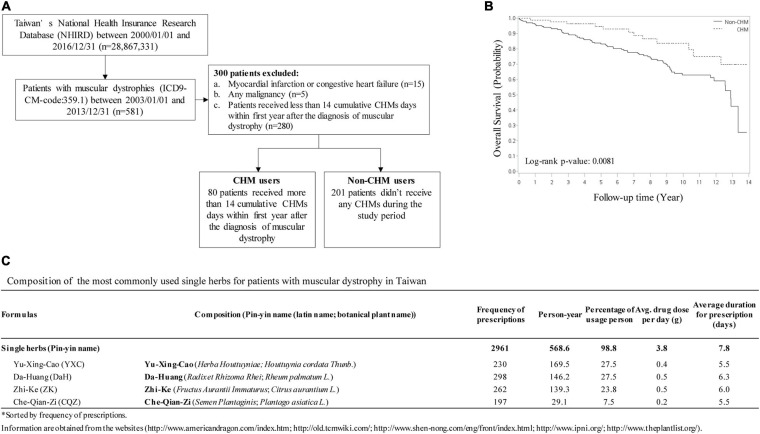
CHM usage among muscular dystrophy patients in Taiwan. **(A)** Selection workflow identifying CHM and non-CHM muscular dystrophic cohort in Taiwan. CHM, Chinese herbal medicine. **(B)** The cumulative incidence of overall survival in CHM users versus non-CHM users. **(C)** Identification of the most commonly used single herbs for patients with muscular dystrophy in Taiwan.

Chinese herbal medicine has been used as an adjunct treatment for muscular dystrophy patients, including DMD, and is offered in Taiwan’s medical healthcare system. To track the outcome of long-term CHM usage, we analyzed and compared non-CHM users to CHM users on overall mortality in patients with muscular dystrophies (Figure 1). A total of 581 patients with muscular dystrophies was identified in the Registry for Catastrophic Illness Patients of Taiwan’s National Health Insurance Research Database (NHIRD^4^) from the National Health Insurance (NHI) program between 2003 and 2013. Among these patients, patients exhibiting myocardial infarction, congestive heart failure, or any other malignancies were excluded (Figure 1A). Total of 80 patients that received more than 14 cumulative CHM days within 1 year after diagnosis were defined as CHM users. Total of 201 patients that received no CHM during the study period were defined as non-CHM users (Figure 1A). Kaplan–Meier survival analysis showed that the cumulative incidence of overall survival was significantly higher in CHM users compared to non-users (*p* = 0.0081, log-rank test). CHM users showed a lower risk of overall mortality than non-CHM users after adjusting for age, gender, prednisolone use, and comorbidities (OR: 0.393, 95% CI: 0.21–0.75, *p* = 0.0044) (Figure 1B). Using association rule mining and network analysis, we identified top 4 single herbs prescribed for the CHM user group: Yu-Xing-Cao (YXC; *Houttuynia cordata Thunb.*), Da-Huang (DaH; *Rheum palmatum L*.), Zhi-Ke (ZK; *Citrus aurantium L.*) and Che-Qian-Zi (CQZ; *Plantago asiatica L.*) (Figure 1C).

### Verification of CHM’s Therapeutic Potential Using DMD hiPSC-Derived Cardiomyocytes

**FIGURE 2:**
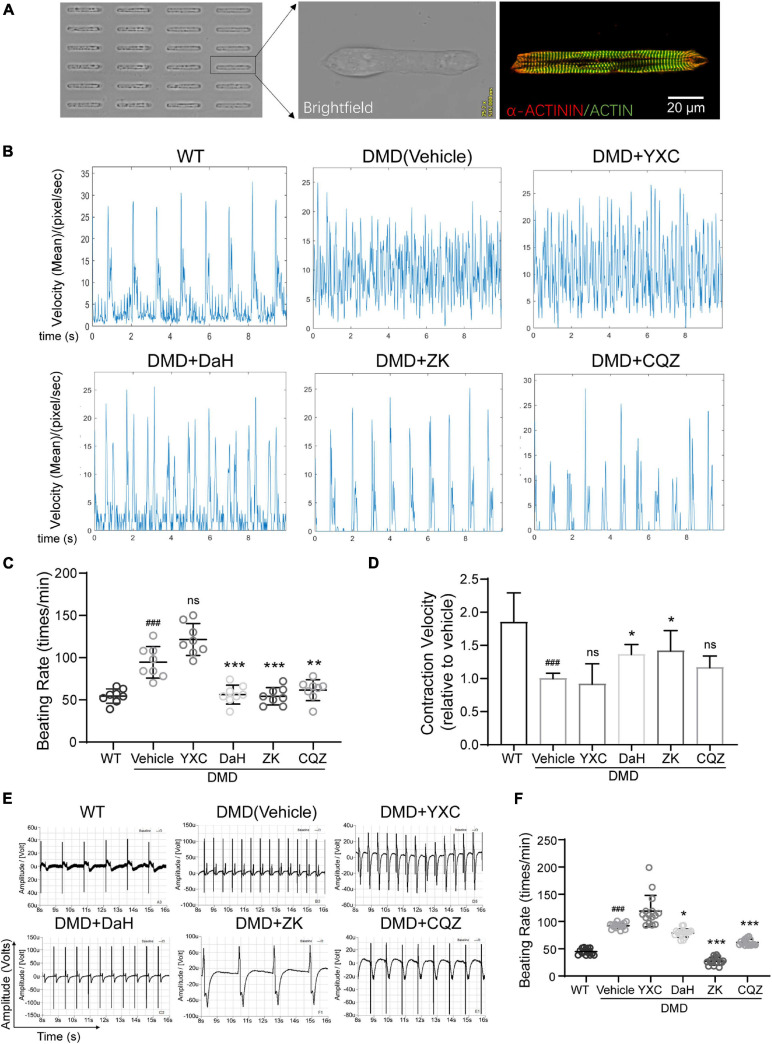
Effect of DaH, ZK, and CQZ treatment on DMD cardiomyocyte function. **(A)** Representative image of micropatterned hiPSC-CMs, showing single cell with aligned sarcomere. **(B)** Representative motion tracing of single wild type (WT) hiPSC-CMs and DMD hiPSC-CMs. **(C)** Beating rate and **(D)** contraction velocity of single DMD hiPSC-CMs treated with YXC, DaH, ZK, CQZ, or Vehicle (*n* = 8). *^###^P* < 0.001 versus WT; *P < 0.05, **P < 0.01, and ***P < 0.001 versus vehicle. **(E)** Representative extracellular field potential (EFP) traces and **(F)** quantification of monolayer DMD hiPSC-CMs treated with YXC, DaH, ZK, CQZ, or Vehicle. WT hiPSC-CMs served as healthy control (*n* = 12). *^###^P* < 0.001 versus WT; *P < 0.05, **P < 0.01, and ***P < 0.001 versus vehicle; ns = no statistical significance.

To verify the effect of these CHMs on DMD cardiomyocyte function, we used DMD hiPSC-CMs. To detected the effect of CHM herbs on single cardiomyocyte contraction, hiPSC-CMs were grown into rod-shape single cells using microprinting (Chang et al., 2020). Micropatterned hiPSC-CMs exhibited well-aligned sarcomere structures after 3 days (Figure 2A). Compared to WT hiPSC-CMs, DMD hiPSC-CMs exhibited aberrant electrophysiology with increased beating rate (Figure 2B). DaH, ZK, and CQZ treatments reduced beating rate in DMD hiPSC-CMs compared to vehicle (Figures 2B,C). Moreover, DaH and ZK treatments increased contraction velocity of DMD hiPSC-CMs (Figure 2D). However, YXC-treated DMD hiPSC-CMs exhibited intensified beating patterns, suggesting cardiotoxicity (Figures 2B–D). Next, we evaluated the effects of the four CHM herbs on cardiac electrophysiology using hiPSC-CM monolayers on the Nanion CardioExcyte96 platform (Figure 2E). Similar to single cell observations, we observed reduced beating rate in DMD hiPSC-CMs treated with DaH, ZK, and CQZ compared to vehicle (Figures 2E,F and Supplementary Figure 1). YXC treatment disrupted electrophysiology evident by aberrant EFP readings, which is in accordance with our single cell results (Figures 2E,F). DaH, ZK, and CQZ treatments significantly strengthened cardiac function (reduced beating rate and increased contraction velocity) in DMD cardiomyocytes. Subsequent experiments were carried out using DaH, ZK, and CQZ.

### Identification of CAT and VCAM1 as Therapeutic Targets for CHM Herbs

**FIGURE 3:**
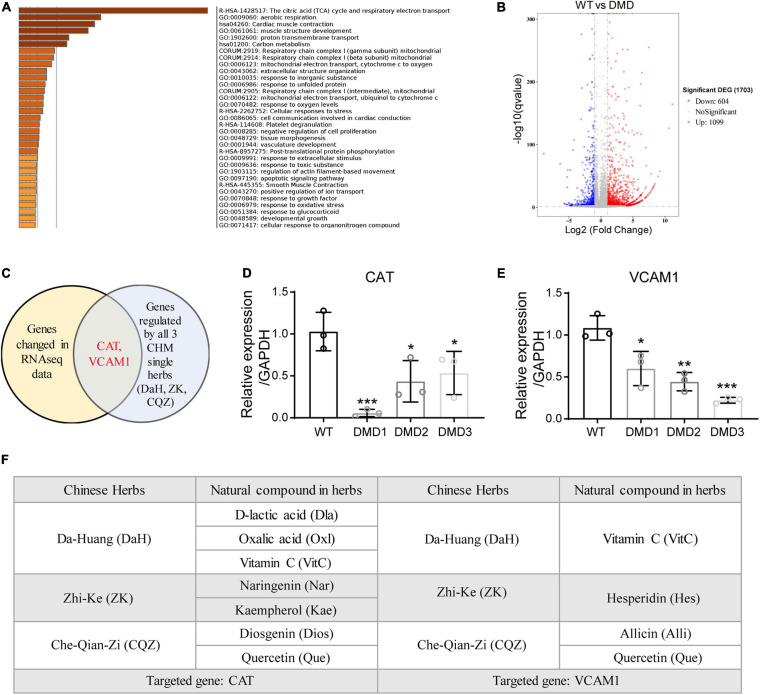
Identification of CAT and VCAM1 as therapeutic targets for DaH, ZK, and CQZ. **(A)** GO term analysis of differentially expressed genes between WT and DMD hiPSC-CMs. Top enrichment clusters were shown, discrete color scale represented statistical significance. **(B)** Volcano plot of differentially expressed genes between WT and DMD hiPSC-CMs. **(C)** Venn diagram showing overlapping genes between differentially expressed gene list and CHM target genes. Relative expression of **(D)** CAT and **(E)** VCAM1 were measured by RT-qPCR (*n* = 3). *P < 0.05, **P < 0.01, and ***P < 0.001. **(F)** Summary of active compounds in DaH, ZK, and CQZ targeting CAT and VCAM1, respectively.

To identify the active compounds in the three CHM herbs, we performed transcriptomic profiling of control and DMD hiPSC-CMs and cross-referenced differentially expressed genes with CHM active compound databases (SuperTCM, SymMap, TCMSP). Whole-transcriptome RNA sequencing analysis identified a total of 1099 upregulated and 604 downregulated differentially expressed genes in DMD hiPSC-CMs compared to WT hiPSC-CMs (Figure 3A). Gene ontology analyses revealed that genes involved in TCA cycle and mitochondrial electron transport, cardiac muscle contraction, and structure development were significantly affected in DMD hiPSC-CMs (Figure 3B). Next, we downloaded active compounds and corresponding gene targets of our CHM herbs from databases mentioned above. Using cross-comparison of transcriptomic profiling data and CHM database, we identified CAT and VCAM1 as major targets for our three CHM herbs (Figure 3C). Using RT-qPCR, we confirmed that CAT and VCAM1 are downregulated in DMD hiPSC-CMs compared to control hiPSC-CMs (Figures 3D,E). Next, we sought to determined which active compounds targeted CAT or VCAM1 in DMD hiPSC-CMs. Active compounds targeting CAT in DA-Huang included: Oxalic acid (Oxl), Vitamin C (VitC), D-lactic acid (Dla); in Zhi-Ke included: Naringenin (Nar), Kaempferol (Kae); and in Che-Qian-Zi included: Diosgenin (Dios), Quercetin (Que) (Figure 3F). Active compounds targeting VCAM1 included: Vitamin C (VitC) in Da-Huang; Hesperidin (Hes) in Zhi-Ke; Quercetin (Que) and Allicin (Alli) in Che-Qian-Zi (Figure 3F).

### Single Herbs Decreased ROS Accumulation Through Decomposing H_2_O_2_ and Improved Structure of DMD hiPSC-CMs

**FIGURE 4:**
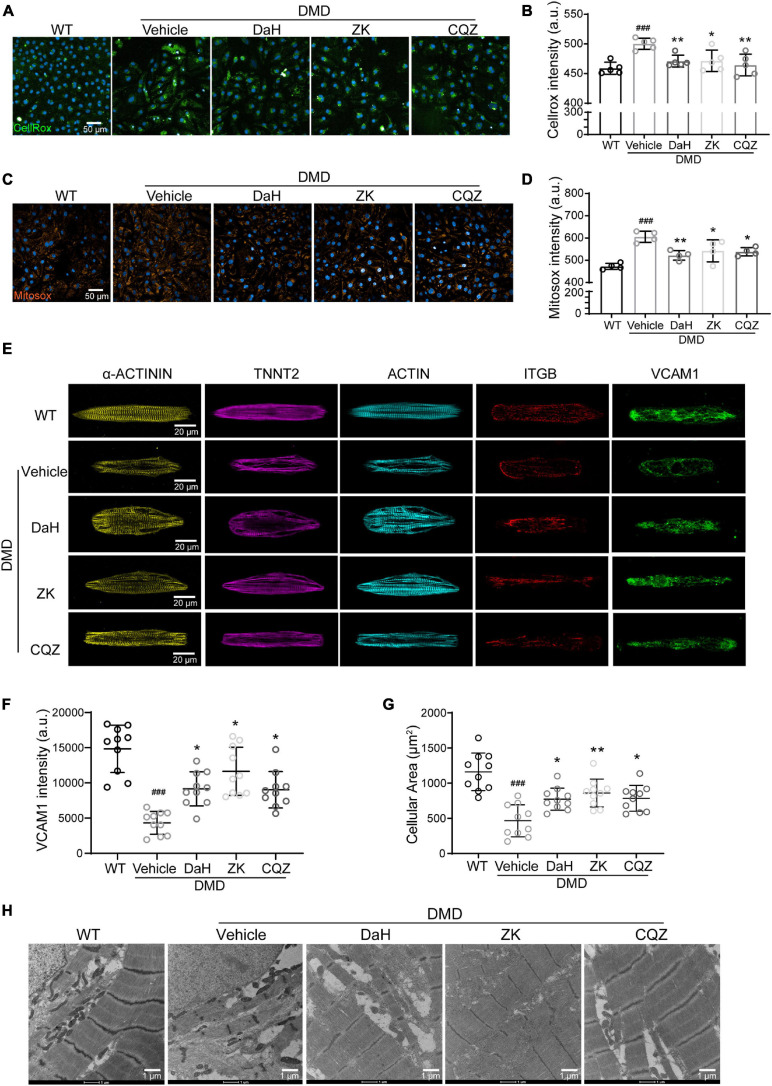
DaH, ZK, and CQZ treatments decreased ROS accumulation and improved cytoskeletal structure of DMD hiPSC-CMs. **(A)** Representative micrographs of WT and DMD hiPSC-CMs treated with DaH, ZK, CQZ or vehicle stained for cellular ROS (Cellrox). **(B)** Intracellular ROS levels were determined by measuring the level of fluorescent Cellrox (*n* = 5). *^###^P* < 0.001 versus WT; *P < 0.05, **P < 0.01 versus vehicle. **(C)** Representative micrographs of WT and DMD hiPSC-CMs treated with DaH, ZK, CQZ or vehicle stained for mitochondrial ROS (Mitosox). **(D)** Mitochondrial ROS levels were determined by measuring the level of fluorescent Mitosox (*n* = 4). *^###^P* < 0.001 versus WT; *P < 0.05, **P < 0.01 versus vehicle. **(E)** Immunofluorescence micrographs of focal adhesion related proteins. **(F)** Quantification of total VCAM1 levels and **(G)** cell size of WT and DMD hiPSC-CMs (*n* = 10). *^###^P* < 0.001 versus WT; *P < 0.05, **P < 0.01 versus vehicle. **(H)** Representative transmission electron microscopy images of WT and DMD hiPSC-CMs.

Catalase plays a major role in protecting the adverse effects of accumulating peroxides via hydrogen peroxide (H_2_O_2_) hydrolysis (Dai et al., 2009). We used intracellular ROS and H_2_O_2_ levels as a readout for CAT activity. Compared with WT hiPSC-CMs, DMD hiPSC-CMs showed increased ROS levels at baseline (Figures 4A,B). However, we observed a drastic decrease in intracellular ROS in DMD hiPSC-CMs treated with DaH, ZK, and CQZ (Figures 4A,B). Similarly, DaH, ZK and CQZ treatment also reduced mitochondrial superoxide levels (Figures 4C,D) in DMD hiPSC-CMs compared to vehicle. Together, these results show that DaH, ZK, or CQZ alone was able to reduce ROS burden in DMD hiPSC-CMs.

Focal adhesion is dynamic and integrins aggregates are formed to promote cellular adhesion by strengthening the connection between cytoskeleton and extracellular matrices. Vascular cell adhesion molecule 1 (VCAM1), located at cell membrane, plays an important role in the binding of integrin beta 1 (ITGB). To evaluate the impact of CHM herbs on myofilament structure of DMD hiPSC-CMs, we performed immunofluorescence staining on CHM-treated DMD hiPSC-CMs. At baseline, DMD hiPSC-CMs exhibited decreased VCAM1 protein distribution compared with WT hiPSC-CMs (Figures 4E,F). Treatment with DaH, ZK, and CQZ increased VCAM1 expression and protein levels in DMD hiPSC-CMs significantly (Figures 4E,F and Supplementary Figure 2B). Interestingly, reduced VCAM1 levels were accompanied with reduced cardiomyocyte surface area in DMD hiPSC-CMs and treatment with DaH, ZK, and CQZ reversed this phenotype (Figure 4G). Using transmission electron microscopy, DaH, ZK, and CQZ treated DMD hiPSC-CMs exhibited wider sarcomere structures compared to untreated DMD hiPSC-CMs (Figure 4H). These data suggest that DaH, ZK, and CQZ treatment improves DMD cardiomyocyte ultrastructure through induction of VCAM1 protein.

### CAT Targeting Compounds Protect DMD Cardiomyocytes From Oxidative Damage and Rescue Cardiomyocyte Function

**FIGURE 5:**
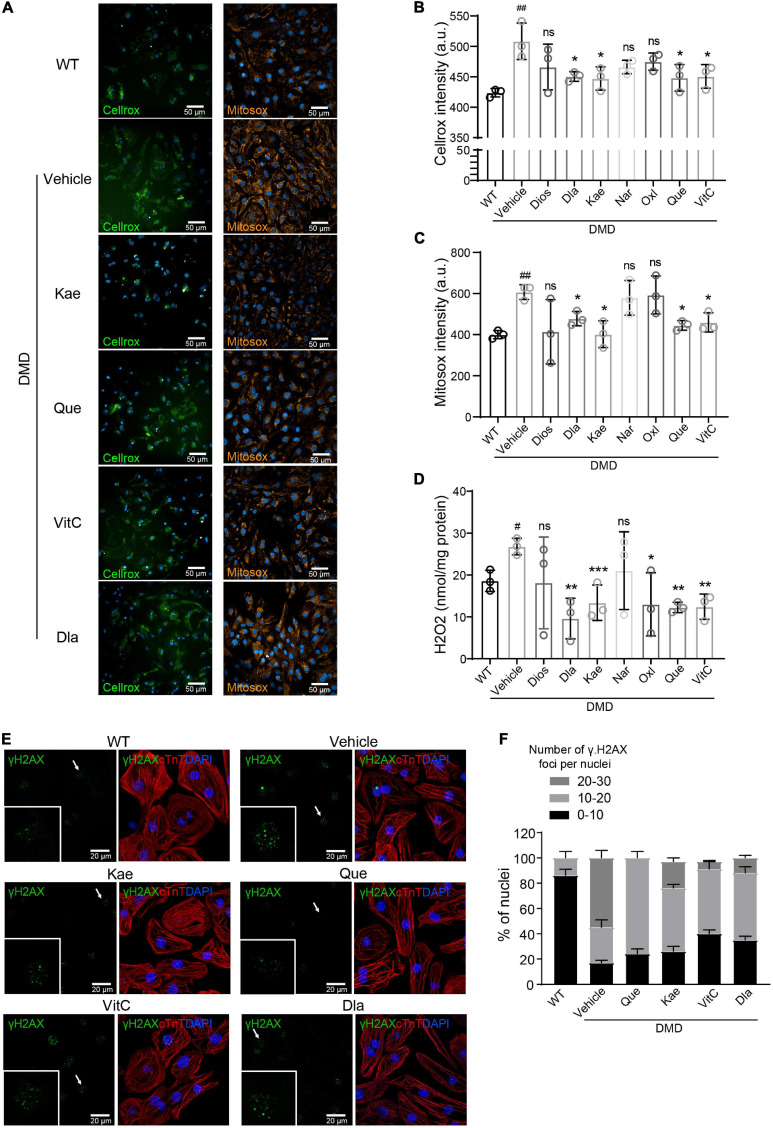
Active compounds from DaH, ZK, and CQZ decreased intracellular ROS levels and protect cells from oxidative DNA damage. **(A)** Representative micrographs of WT and DMD hiPSC-CMs treated with active compounds or vehicle stained for cellular ROS (Cellrox) and mitochondrial ROS (Mitosox). **(B)** Intracellular ROS levels (Cellrox), **(C)** mitochondrial ROS levels (Mitosox), and **(D)** H_2_O_2_ levels in WT and DMD hiPSC-CMs were measured (*n* = 3). *^#^P* < 0.05, ^#^*^#^P* < 0.01 versus WT; *P < 0.05, **P < 0.01, and ***P < 0.001 versus vehicle; ns = no statistical significance. **(E)** Representative images of γH2A.X staining in WT and DMD hiPSC-CMs treated with active compounds or vehicle, only active compounds showing statistical significance were shown. **(F)** Quantification of percentage of γH2A.X foci per nucleus in hiPSC-CMs.

To test the antioxidant efficacy of the seven single natural compound targeting CAT, we measured intracellular ROS levels using Cellrox and Mitosox (Figure 5A). Compared to vehicle treatment, Kae, Que, VitC, and Dla treatment significantly decreased total ROS accumulation in DMD hiPSC-CMs while Dios, Nar, Oxl only showed a trend (Figures 5A,B). Production of mitochondrial superoxide was reduced when DMD hiPSC-CMs were treated with Kae, Que, VitC, and Dla but not Dios, Nar, and Oxl (Figures 5A,C). Kae, Que, VitC, Oxl, and Dla treatment also showed decrease in H_2_O_2_ level (Figure 5D), which is in agreement with our cellular ROS results (Figures 5A,B). Compared to vehicle treated DMD hiPSC-CMs, Que, Kae, VitC, and Dla treatments decreased γH2AX foci accumulation in nuclei (Figures 5E,F), a readout for DNA damage.

**FIGURE 6:**
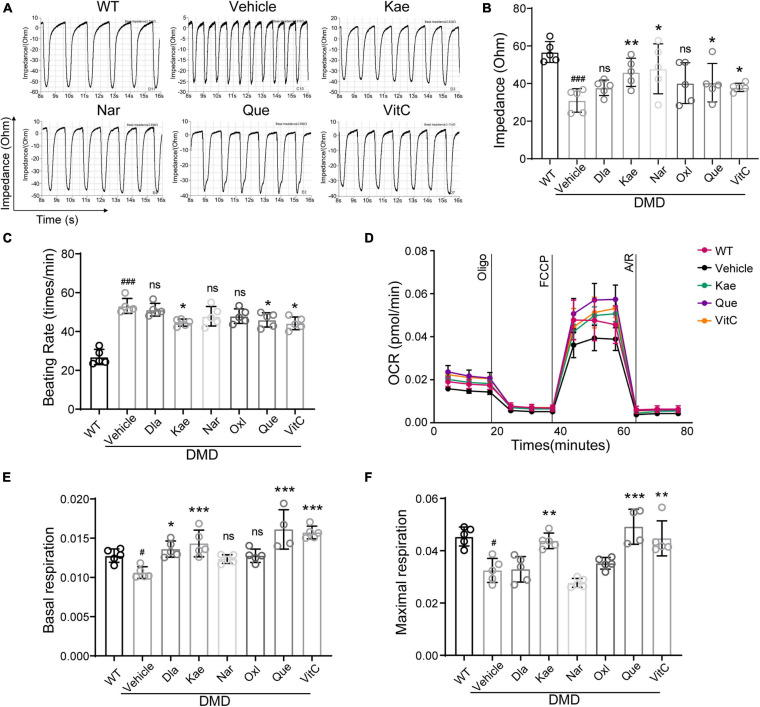
Active compounds from DaH, ZK, and CQZ increased DMD hiPSC-CMs contractility and mitochondrial respiration. **(A)** Representative impedance (IMP) traces of monolayer DMD hiPSC-CMs treated with active compounds or vehicle. **(B)** Quantification of the IMP and **(C)** beating rate of monolayer DMD hiPSC-CMs treated with active compounds or vehicle. WT hiPSC-CMs served as healthy control. **(D)** Real-time mitochondrial respiration measurements of hiPSC-CMs. **(E)** Quantification of basal mitochondrial respiration and **(F)** maximal respiration of WT and DMD hiPSC-CMs treated with active compounds or vehicle (*n* = 5). ^#^*P* < 0.05 versus WT; *^###^P* < 0.001 versus WT; *P < 0.05, **P < 0.01, and ***P < 0.001 versus vehicle; ns = no statistical significance.

Next, we examined contraction and electrical conduction of DMD hiPSC-CMs using Nanion (Figure 6A). Using cardiomyocyte impedance as a readout of contraction, DMD hiPSC-CMs treated with Que, Kae, VitC, and Nar exhibited a significant increase in contractility compared to vehicle (Figure 6B). Treatment with Que, Kae, and VitC also decreased cell beating rate in DMD hiPSC-CMs compared to vehicle (Figure 6C). It has been demonstrated that high oxidative stress can lead to mitochondrial dysfunction in dystrophic cardiomyocytes (Chang et al., 2016). Among these compounds, Dios showed cytotoxicity (data not shown). Mitochondrial function of DMD hiPSC-CMs treated with the other six compounds were evaluated using Seahorse bioanalyzer (Figure 6D). DMD hiPSC-CMs treated with Que, Kae, VitC, and Dla exhibited higher basal mitochondrial respiration compared to vehicle (Figure 6E); moreover, Que, Kae, and VitC treatments displayed significantly higher maximal respiratory capacity (Figure 6F).

### Structural Fortification via VCAM1 Modulation Improves DMD hiPSC-CM Contractility

**FIGURE 7:**
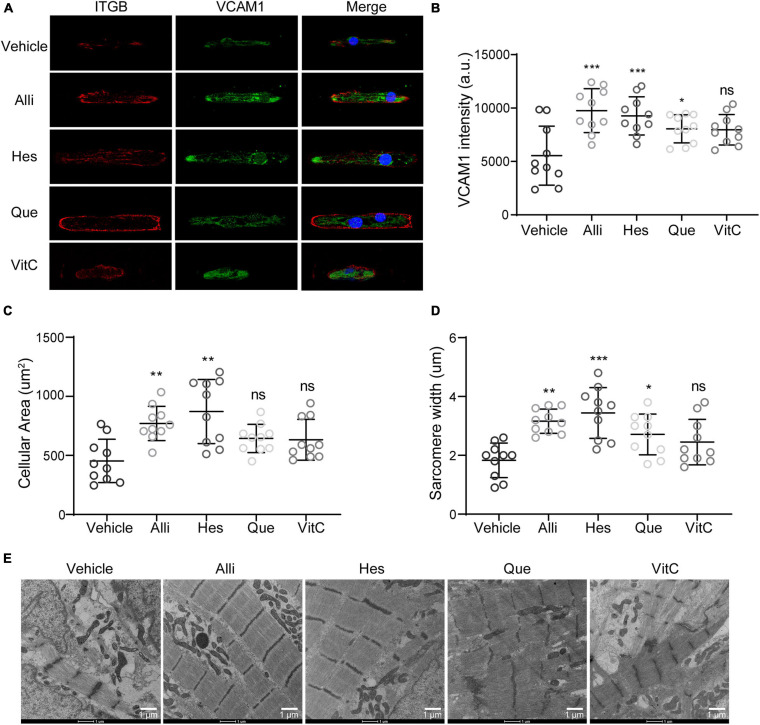
Active compounds from DaH, ZK, and CQZ improved cytoskeletal structure of DMD hiPSC-CMs. **(A)** Representative immunofluorescence micrographs of ITGB and VCAM1 protein in DMD hiPSC-CMs treated with active compounds. Quantification of **(B)** VCAM1 levels, **(C)** cell size, and **(D)** sarcomere lengths of DMD hiPSC-CMs treated with Alli, Hes, Que, VitC, or vehicle (*n* = 10). *P < 0.05, **P < 0.01, and ***P < 0.001; ns = no statistical significance. **(E)** Representative transmission electron microscopy images of DMD hiPSC-CMs treated with Alli, Hes, Que, VitC, or vehicle.

Besides ROS modulation through CAT, our analysis also identified VCAM1 targeting compounds. To evaluate the impact of four natural compounds targeting VCAM1, we performed immunofluorescence stainings for ITGB and VCAM1protein in hiPSC-CMs (Figure 7A). Compared to vehicle treatment, we observed an increase in VCAM1 protein levels when DMD hiPSC-CMs were treated with Alli, Hes, and Que (Figures 7A,B). Moreover, cell size and sarcomere lengths were increased after Alli/Hes and Alli/Hes/Que treatment, respectively (Figures 7C,D). Using transmission electron microscopy, Alli, Hes, and Que treated DMD hiPSC-CMs exhibited wider sarcomere structures compared to vehicle (Figure 7E). In conclusion, the results exhibited that Allicin and Hesperidin play an obvious role in the morphology maintain of DMD hiPSC-CMs.

### Overexpression of CAT or VCAM1 Restores Mitochondrial Function, Contractility, and Structural Integrity in DMD hiPSC-CMs

**FIGURE 8:**
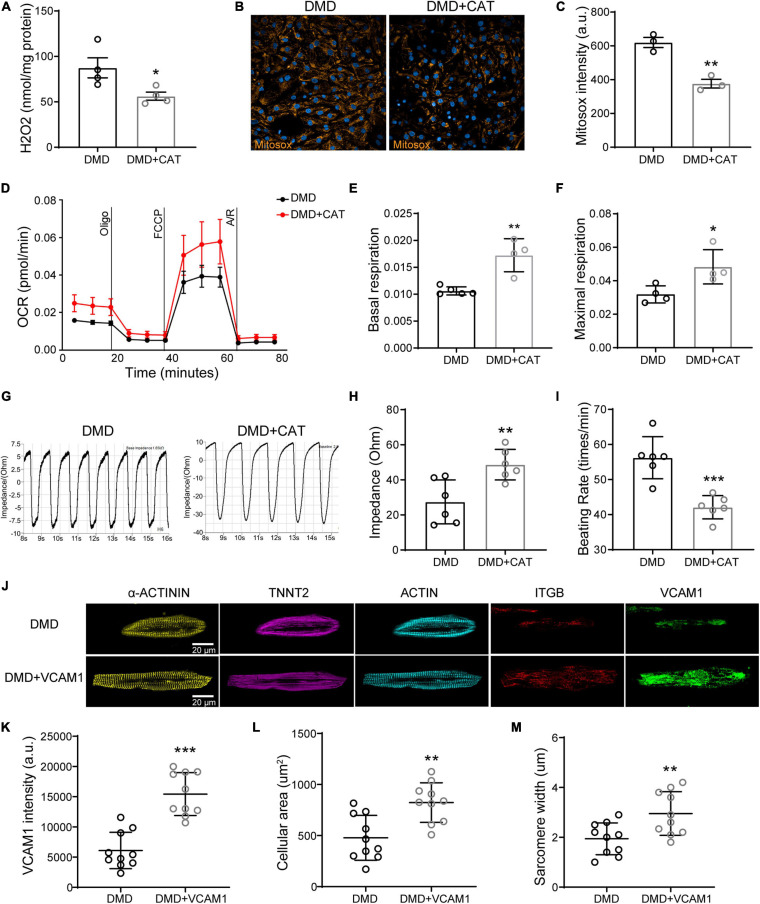
Overexpression of CAT or VCAM1 in DMD hiPSC-CMs. **(A)** H_2_O_2_ levels, **(B)** Mitosox, and **(C)** mitochondrial ROS levels were quantified in DMD hiPSC-CMs overexpressing CAT. **(D)** Mitochondrial respiration was measured in CAT overexpressing DMD hiPSC-CMs. **(E)** Quantification of basal respiration and **(F)** maximal respiration. **(G)** Representative impedance traces of monolayer DMD hiPSC-CMs treated with CAT lentivirus or control and quantification of the **(H)** impedance and **(I)** beating rate (*n* = 6). **P < 0.01 and ***P < 0.001. **(J)** Immunofluorescence micrographs of focal adhesion related proteins and VCAM1. Quantification of **(K)** VCAM1 levels, **(L)** cell size, and **(M)** sarcomere length in DMD hiPSC-CMs treated with CAT lentivirus or control (*n* = 10). *P < 0.05, **P < 0.01, and ***P < 0.001.

To show cause and effect in improving myocardial function, we overexpressed CAT or VCAM1 in DMD hiPSC-CMs (Supplementary Figure 3). CAT overexpression significantly reduced intracellular H_2_O_2_ level (Figure 8A) and mitochondrial superoxide (Figures 8B,C) in DMD hiPSC-CMs compared to untreated. Compared to vehicle control, CAT overexpressing DMD hiPSC-CMs exhibited increased basal mitochondrial respiration and maximal respiration compared to untreated, respectively (Figures 8D–F). By measuring impedance as a readout of contractility, we found that CAT induction improved DMD hiPSC-CMs contractility and decreased beating rate (Figures 8G–I). Similar to active compound results, VCAM1 overexpression (Figures 8J,K) resulted in increased cell size and sarcomere lengths of DMD hiPSC-CMs (Figures 8L,M). Together, our results show that restoration of either CAT or VCAM1 significantly lowers ROS levels and improves cardiac contractility, mitochondrial respiration, and sarcomere structure.

## Discussion

Clinically, DMD patients succumb to dilated cardiomyopathy and respiratory complications at second or third decade of life. However, our treatment options for DMD patients are still very limited. Although corticosteroids can slow down muscle loss in DMD patients, weight gain and bone fracture risks often drive patients into non-compliance. Despite muscle and respiratory interventions have significantly increased the life expectancy of DMD patients, however, dilated cardiomyopathy is now the leading cause of death for DMD patients (Spurney, 2011). Results of AAV and micro-dystrophin clinical trials showed significant increase in micro-dystrophin expression but very limited improvement in mobility as per the NAAA evaluation (Mendell et al., 2020). Balancing viral dosage and DMD protein expression remains a challenge for DMD gene therapy. Treatment using autologous or allogeneic stem cells injection remains to be translated (Meregalli et al., 2013). Therefore, there is an urgent need for better biomarker monitoring and alternative treatment options for DMD patients.

Patients induced pluripotent stem cells derived cardiomyocytes (hiPSC-CMs) offer a scalable platform that can be used for drug screening and validation (Sallam et al., 2014). Availability of DMD cardiomyocytes enables us to study DMD cardiac functions such as electrophysiology and contractility. By cross-comparing clinical, RNAseq and CHM databases, we used DMD hiPSCs-derived cardiomyocytes as a surrogate to screen for active compounds in CHM prescription. Based on our multi-omics analysis, we identified and validated CAT and VCAM1 as novel targets for treating DMD cardiomyopathy.

Catalase is a major antioxidant enzyme which hydrolyze hydrogen peroxide (H_2_O_2_) into water and O_2_. H_2_O_2_ is an ubiquitous ROS in biological systems, which is produced as a byproduct of oxidative metabolism in peroxisomes and mitochondria (Dubreuil et al., 2020). Previous studies showed catalase plays a key role in cellular oxidative balance and cellular redox signaling regulation (Young and Woodside, 2001). Cardiac catalase overexpression has been shown to protect against ROS-induced cell death and oxidant-mediated activation of inflammatory signaling pathway (Dai et al., 2009; Otera and Fujiki, 2012; Cong et al., 2015). Catalase has also been shown to restore cardiac contractile dysfunction and intracellular Ca^2+^ mishandling induced by ethanol (Zhang, 2003) or LPS (Turdi et al., 2012). In our study, we found that DaH, ZK, and CQZ could protect DMD hiPSC-CMs from oxidative damage, improve mitochondrial functions, and cell contraction. Based on our multi-omics analysis, we further validated these finding by validating active compounds as well as CAT and VCAM1 overexpression.

Sarcomere integrity is critical to cardiomyocytes structural adaptation during cell development and disease process. Dystrophin deficiency results in calcium overload, increased reactive oxygen species, and mitochondrial dysfunction (Gonzalez et al., 2014; Chang et al., 2016). Cardiomyocytes continuously produce moderate levels of ROS and multiple antioxidant systems are needed to maintain a redox homeostasis (Santos et al., 2011; Bertero and Maack, 2018). In absence of dystrophin, stretch induced NOX2 increases ROS burden in cardiomyocytes (Prosser et al., 2011) and skeletal muscles (Petrillo et al., 2017). Mechanical distortion during physiologic stretch in muscles would induce NOX2-dependent ROS production via an intact microtubule network (Prosser et al., 2011; Khairallah et al., 2012). Given full-length dystrophin restoration is yet to be realized, drugs targeting ROS and/or promoting structure support could potentially slow down DMD progression.

Here, we provide evidence where CAT and VCAM1 are two key genes downregulated in DMD cardiomyocytes. By leveraging on clinical prescription and outcome data, we identified three CHM herbs that lowered ROS levels, increased mitochondrial respiration, reduced beating frequency, increased contraction velocity, and boosted structural integrity in dystrophin-deficient cardiomyocytes. Further, we identified and showed active compounds that targeted CAT and VCAM1 provided equal efficacy. Lastly, we demonstrate that overexpression of CAT or VCAM1 conferred similar benefits. Together, our data identifies CAT and VCAM1 as novel targets and demonstrate that increase in activity or protein levels can restore cardiac function in DMD cardiomyocytes. We also demonstrate the feasibility in using clinical data with transcriptomic data to identify and validate new drug candidates using hiPSC-CM technology.

## Data Availability Statement

The datasets presented in this study can be found in online repositories. The names of the repository/repositories and accession number(s) can be found below: https://www.ncbi.nlm.nih.gov/geo/query/acc.cgi?acc=GSE166560.

## Author Contributions

BL, Y-JL, and AC conceived the research and contributed to the writing of the manuscript. BL and WX performed the research and analyzed the data. W-ML and J-SC contributed to the analysis of clinical data in Taiwan. All the authors contributed to the article and approved the submitted version.

## Conflict of Interest

The authors declare that the research was conducted in the absence of any commercial or financial relationships that could be construed as a potential conflict of interest.

## Footnotes

1 http://bioinf-applied.charite.de/supertcm/

2 https://www.symmap.org/

3 https://tcmspw.com/tcmsp.php

4 http://nhird.nhri.org.tw/
