# Supplementary material for: Targeting of CAT and VCAM1 as Novel Therapeutic Targets for DMD Cardiomyopathy
Source: Front Cell Dev Biol. 2021 Apr 1;9:659177. doi: 10.3389/fcell.2021.659177 (PMC8047121; doi:10.3389/fcell.2021.659177)
Supplement: Supplementary file 1 [file Data_Sheet_1.PDF]

SUPPLEMENTARY MATERIAL

Targeting of CAT and VCAM1 as novel therapy for DMD cardiomyopathy

Supplementary Tables

Supplementary Table S1. Information for DMD-hiPSC lines.

Patient hiPSC lines						
Classification	hiPSC lines	Mutation	Age	Tissue	Gender	Source
Healthy	Con#	Isogenic control of DMD1	16	Urine mesenchymal	male	Universityof Washington
DMD	DMD1	DMD (c.19delG)	16	Urine mesenchymal	male	University of Washington
DMD	DMD2	DMD (c.9204_9207del)	9	PBMC	male	Stanford
DMD	DMD3	DMD (c.3638_3650del)	6	PBMC	male	Stanford

Supplementary Table S2. Real-Time quantitative PCR Primers.

Primer	Forward	Reverse
CAT	TGGGATCTCGTTGGAAATAACAC	TCAGGACGTAGGCTCCAGAAG
VCAM1	TTTGACAGGCTGGAGATAGACT	TCAATGTGTAATTTAGCTCGGCA
GAPDH	GGAGCGAGATCCCTCCAAAAT	GGCTGTTGTCATACTTCTCATGG

Supplementary Table S3. Cross-comparison results of DMD hiPSC-CMs transcriptomic profiling and CHM databases.

Zhi-Ke (ZK)	Che-Qian-Zi (CQZ)	Da-Huang (DaH)
CAT	CAT	CAT
VCAM1	VCAM1	VCAM1
NDUFAF1	HSPA5	LPL
	SPP1	RENBP
	CLDN4	SLC2A4

	FABP3	ALB
	SLC2A4	DDIT3
		GABRP
		HSPA5

Supplementary Figures and Figure Legends

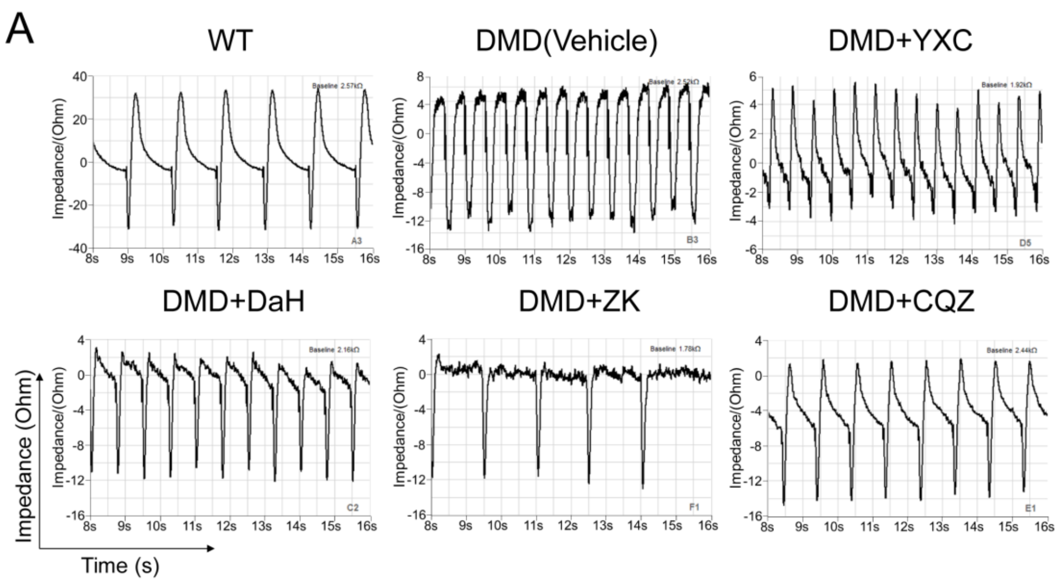

Supplementary Figure S1. Expression of CAT and VCAM1 in hiPSC-CMs. (A) Representative impedance (IMP) traces of monolayer DMD hiPSC-CMs treated with YXC, DaH, ZK, CQZ, or Vehicle. WT hiPSC-CMs served as healthy control.

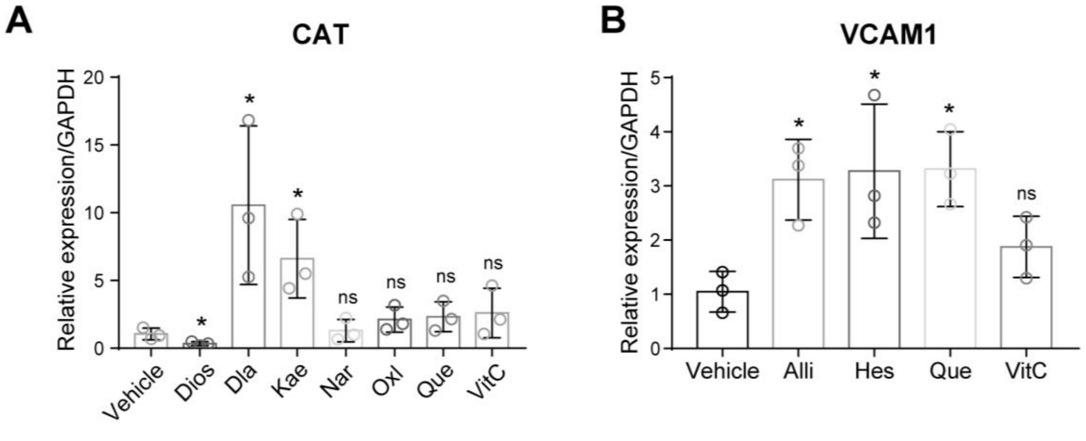

Supplementary Figure S2. Expression of CAT and VCAM1 in hiPSC-CMs. (A) Quantitative PCR detection of CAT and **(B)** VCAM1 mRNA expression in WT and DMD hiPSC-CMs treated with DaH, ZK, CQZ or vehicle. n=3; *$P < 0.05$.

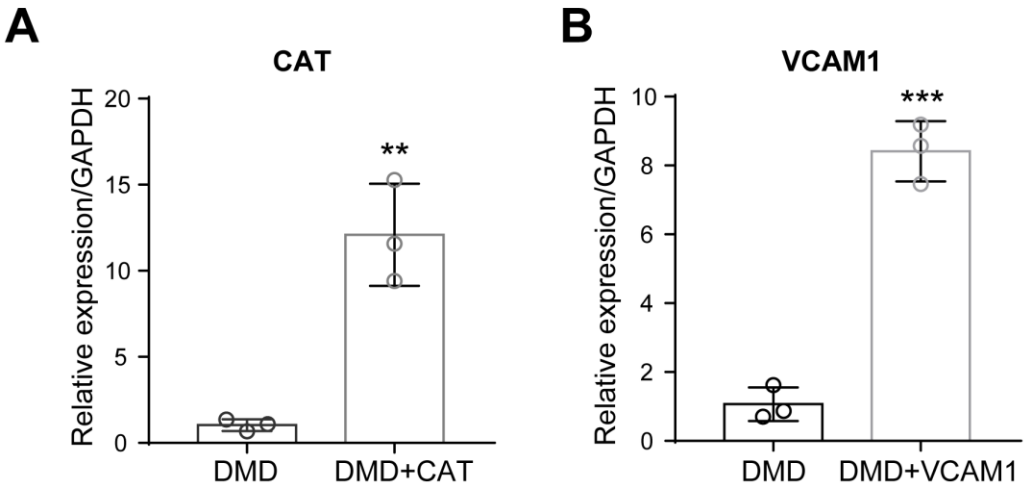

Supplementary Figure S3. Validation of CAT and VCAM1 expression. (A) Quantitative PCR verification of CAT and **(B)** VCAM1 overexpression in DMD hiPSC-CMs (n=3). *$P <$ 0.05, **$P < 0.01$ and ***$P < 0.001$.
